# Engagement in and Benefits of a Short-Term, Brief Psychotherapy Intervention for PTSD During Pregnancy

**DOI:** 10.3389/fpsyt.2022.882429

**Published:** 2022-06-15

**Authors:** Sara L. Kornfield, Rachel L. Johnson, Liisa V. Hantsoo, Rachel B. Kaminsky, Rebecca Waller, Mary Sammel, C. Neill Epperson

**Affiliations:** ^1^Penn Center for Women's Behavioral Wellness, Department of Psychiatry, Perelman School of Medicine, University of Pennsylvania, Philadelphia, PA, United States; ^2^Department of Biostatistics and Informatics, Colorado School of Public Health, University of Colorado, Denver, CO, United States; ^3^Department of Psychiatry and Behavioral Sciences, School of Medicine, Johns Hopkins Medicine, Baltimore, MD, United States; ^4^Psychology Department, University of Pennsylvania, Philadelphia, PA, United States; ^5^Department of Psychiatry, School of Medicine, University of Colorado Anschutz Medical Campus, Aurora, CO, United States; ^6^Department of Family Medicine, School of Medicine, University of Colorado, Aurora, CO, United States

**Keywords:** perinatal mental health, PTSD—post-traumatic stress disorder, brief interventions, women's health, ACE

## Abstract

Trauma-related symptoms and post-traumatic stress disorder (PTSD) are common during pregnancy and have adverse effects on pregnancy and birth outcomes, post-partum maternal mental health, and child development. The arousal symptoms associated with PTSD, including heightened or dysregulated physiology, may contribute to these adverse outcomes. Low-income minoritized women may be at highest risk given more lifetime exposure to trauma and limited access to mental health care. While evidence-based psychotherapies for PTSD exist, none are targeted to non-treatment seeking individuals nor specifically integrated with prenatal care. Thus, we developed and tested the efficacy of a short-term (four sessions) brief (30–45 min) psychotherapeutic intervention designed to address PTSD symptoms in pregnant women receiving prenatal care at two urban medical centers. Participants were 32 pregnant women with an average gestational age of 18.5 weeks at the time of enrollment. The sample was overwhelmingly non-Caucasian, single, and reported very low income. Participants completed measures of trauma-related symptoms (Post-traumatic Stress Disorder Checklist, PCL), and depression (Edinburgh post-natal Depression Scale, EPDS) at baseline, twice during treatment, post-treatment, and at 10–14 weeks post-partum. The intervention was successful at significantly decreasing symptoms of PTSD (PCL score = −20.27, 95% CI: −25.62, −14.92, *P* < 0.001, W = −7.43) and depression (EPDS score = −4.81, 95% CI: −7.55, −2.06, *P* = 0.001, W = −3.23) by the final session. These benefits were sustained at post-treatment and post-partum follow ups. Future research should further explore the effectiveness of this treatment in a randomized controlled trial.

## Introduction

Post-traumatic stress disorder (PTSD) is a prevalent public health concern accounting for significant disability (1). In nationally representative samples of non-pregnant individuals, lifetime prevalence rates of PTSD are estimated to be 6.4% (2) and those of partial PTSD (pPTSD; describing a subthreshold version of the disorder, where only some criteria are met, but functional impairment is still evident) (3–5) range from 4 to 11% (6, 7). PTSD rates are even higher for Black people; non-pregnant Black individuals living in violent communities report rates between 30 and 50% (8) and Black pregnant women with a history of trauma exposure report rates of 34–40% (9–12). The prevalence of pPTSD during pregnancy is estimated to be even higher, with 28.6% of women with previous pregnancy complications reporting pPTSD in a subsequent pregnancy (13). Low-income women of color may be especially at-risk for PTSD during pregnancy and less likely than white women to seek or receive treatment (9, 14).

During pregnancy, PTSD is specifically associated with increased risk for both maternal and child morbidity (15) and other adverse outcomes including ectopic pregnancy, (16) spontaneous abortion, (16) and spontaneous preterm birth (17).

Anxiety and stress during pregnancy are associated with poor pregnancy and birth outcomes, including lower birth weights (18–20) and pre-ecclampsia, (21) while active PTSD during pregnancy confers a two-fold risk of preterm delivery (22). *In utero* exposure to maternal psychological distress and anxiety are also related to long-term adverse behavioral sequelae in children, including higher levels of newborn irritability, (23, 24) behavioral dysregulation in preschoolers, (25) and altered hypothalamic pituitary adrenal (HPA) axis function (as measured by cortisol awakening response) among post-adolescent teens (26). In addition to the intergenerational effects of PTSD during pregnancy, (27) low-income minority women with PTSD symptoms during pregnancy are more likely to engage in poor health behaviors, illicit drug and alcohol use, and to have thoughts of self-harm (28). Finally, women with greater lifetime trauma exposure have higher rates of tobacco use during pregnancy, (29) higher rates of premature rupture of membranes, and longer maternal hospital stays (30).

The physiologic underpinnings of PTSD, specifically heightened or dysregulated arousal, may contribute to these adverse pregnancy and birth outcomes. Arousal symptoms are one of the hallmarks of PTSD, with an exaggerated startle response often purported to be one of the cardinal symptoms of the disorder (31). Patients with PTSD have physiological alterations in stress regulation systems, including dysregulation of the HPA axis, which regulates the body's response to threat (32) and can influence the timing of parturition *via* changes in ACTH, cortisol, and CRH (33). Given the high likelihood of the arousal associated with PTSD and pPTSD contributing to adverse pregnancy, birth, and longer-term outcomes, it is therefore critical to investigate methods of delivering high quality evidence-based psychotherapy to trauma-exposed pregnant women.

Existing evidence-based psychotherapies are often recommended for individuals suffering from PTSD (34). However, non-treatment seeking patients suffering from PTSD during pregnancy may not find the recommended gold-standards in evidence-based treatments for PTSD to be acceptable or realistic, especially since these can entail up to 16 sessions of Prolonged Exposure Therapy or Cognitive Processing Therapy lasting 60–90 min each. Similar observations have been made in non-pregnant samples, which has resulted in more efforts to integrate mental health care in general into primary care settings (35). While the focus has traditionally been on treating depression and anxiety in primary care settings, (36) PTSD treatment in these settings is gaining traction (37). Recently, prolonged exposure for primary care (PE-PC), a four 30-min session treatment, has been piloted in both military and civilian samples and shown to be effective at reducing PTSD, depression, and related mental health symptoms (38, 39). Similarly, a small recent study of a four-session narrative exposure based treatment for prenatal PTSD suggested acceptability to patients and clinically meaningful change on PTSD symptoms (40). These findings suggest that short term and brief treatments may be warranted in order to be acceptable to non-treatment seeking individuals, or those experiencing less severe symptoms. In addition, the evidence base for specifically treating pPTSD, including during pregnancy, is only now in development (40). Brief and shorter-term treatments for PTSD and pPTSD focused on arousal symptoms may be especially important in pregnant populations given the potential for adverse pregnancy outcomes due to untreated illness, and concerns for intergenerational transmission of trauma (27, 41). Despite the established link between prenatal PTSD and complications of pregnancy, childbirth, and long term sequalae for mothers and children, several recent reviews suggests that research on the diagnosis and treatment of prenatal PTSD and other trauma-related disorders is extremely limited (40, 42).

To address this need, we developed and applied a novel short-term and brief psychotherapy intervention focused on psychoeducation about PTSD and pPTSD symptoms, behavior activation as a proxy for behavioral exposure, and mindfulness to address arousal symptoms to non-treatment seeking pregnant women attending a Medicaid-serving prenatal care clinic. Based on clinical records and a survey of women receiving prenatal care in our clinic, (43) we noted that women attend an average of four psychotherapy sessions when it is offered in a co-located setting. Thus, we designed our intervention to be delivered in four sessions.

Finally, given that prior life stress and trauma could influence engagement and attendance among non-treatment seeking women, (44, 45) we also aimed to determine how much prior life stress may have contributed to willingness to engage in this treatment. As few studies examine post-partum mental health outcomes beyond 6 weeks post-partum, (46) we investigated the efficacy of our intervention on both short- and longer-term mental health outcomes both during pregnancy and 10–14 weeks post-partum.

## Methods

### Subject Recruitment and Screening

All recruitment took place within hospital based prenatal care clinics in the Penn Medicine Health Care System (University of Pennsylvania) and the Barnes Jewish Hospital System affiliated with the Washington University in St. Louis School of Medicine between July 2014 and September 2019. Consecutive pregnant women between 12^0^ and 22^6^ weeks gestation were screened for eligibility for the parent study and invited to participate in the treatment study if they met criteria for PTSD or pPTSD. This window in the second trimester was chosen for enrollment to allow for a pregnancy to be established, to limit the risk of enrolling women very early in gestation who may miscarry, and to allow for sufficient time during gestation to schedule and attend the six visits required during the pregnancy. Inclusion criteria required participants to be between ages 18–39, able to speak and read English at a 6th grade level, currently pregnant (up to 22^6^ weeks gestation), and meet DSM-IV-TR diagnostic criteria for PTSD or pPTSD. For the purposes of this study, pPTSD was defined as meeting symptom criteria for clusters A (trauma), B (re-experiencing), and C (avoidance) or A, B, and D (arousal) (47). Because our focus was on arousal symptoms, if patients met criteria for pPTSD with symptoms from A, B, and C, we required that they also had at least one arousal symptom from cluster D. All participants were without serious medical condition, known fetal anomaly, use of psychotropic medication or substances of abuse for at least 6 months, or lifetime history of a psychotic disorder. This study was approved by the Institutional Review Boards of both the University of Pennsylvania and the Washington University in St. Louis.

### Assessment Overview

After providing written informed consent, all participants were administered the Structured Clinical Interview for Diagnosis (SCID) DSM-IV, The Perceived Stress Scale (PSS), the Edinburgh Post-natal Depression Scale, (EPDS), the Post-traumatic Stress Disorder Checklist (PCL-IV), the Adverse Childhood Experiences Questionnaire (ACE), and the TLEQ (Traumatic Life Experiences Questionnaire). Those who met study criteria for PTSD or pPTSD were invited to take part in the brief psychotherapy intervention.

#### Perceived Stress Scale

The PSS assesses how unpredictable, uncontrollable, overloaded, and stressful they perceive their life to be (48). On 10 questions related to perceived stress, participants indicated how often they felt or thought a certain way over the last month (0 = never, 1 = almost never, 2 = sometimes, 3 = fairly often, 4 = very often). PSS scores were calculated by summing all items.

#### Edinburgh Post-natal Depression Scale

The EPDS assesses depressive symptomatology over the past week (49). Although the scale was originally developed to measure depressive symptoms in post-partum women, it has been validated for use in antepartum women as well (50, 51). On 10 items measuring depressive symptoms, participants indicated how often they felt or thought a certain way, e.g., “I have been sad or miserable” on a scale from 0 to 3, with higher scores indicating greater frequency of depressive symptoms. EPDS scores were calculated by summing all items.

#### Post-traumatic Stress Disorder Checklist-DSM IV

The PCL-4 is a standardized self-report rating scale for PTSD that comprises 17 items that correspond to the DSM-IV-TR version of PTSD symptoms (52). For the purposes of this study we used the civilian version of the PCL which can be applied to any traumatic event. Respondents indicate how much they have been bothered by a particular symptom over the past month using a 5-point scale ranging from 1 Not at all to 5 Extremely.

#### Adverse Childhood Experiences Questionnaire

The ACE-Q assesses 10 types of adversity experienced before age 18: abuse (emotional, physical, and sexual), neglect (emotional and physical), and household dysfunction (parental separation or divorce, household domestic violence, household substance abuse, parental mental illness, member of household imprisoned) (53). Each exposure was counted as one point. ACE scores were computed by summing up all exposures (0–10).

#### Traumatic Life Events Questionnaire

The TLEQ assesses whether an individual has been exposed to events which are conceptualized in the DSM-IV as being sufficiently aversive to result in a feeling of helplessness, horror or fear and thus qualify as a trauma (54). The TLEQ measures exposure to 23 such events. Items are summed for a total exposure count.

### Intervention Procedure

Traditionally, primary care-mental health treatments (for depression) are brief and problem-focused, compared to specialized psychological interventions in which longer session durations and frequency are the norm (55, 56). This is an important consideration for pregnant women who are operating on a deadline of the pending birth and for whom symptom resolution may be important for pregnancy, birth, and long-term health outcomes.

The newly developed psychotherapeutic intervention used in this study, consisted of four 30–40 min sessions targeted at psychoeducation, cognitive restructuring, behavior activation, and mindfulness. Our brief psychotherapy builds upon a treatment devised for delivery in primary care settings and is meant to be convenient for patients and cost effective for practitioners. It uses components from cognitive behavior therapy (CBT), (57) and Mindfulness Based Stress Reduction (MBSR) (58). Sessions were delivered weekly and the research team allowed participants to make up missed sessions at any point prior to delivery.

*Session 1* focuses on gathering information about the patient's symptoms, developing rapport, and providing education about PTSD symptoms. Discussing common symptoms and typical reactions to trauma can assist the patient in recognizing how these symptoms may be disruptive to her functioning. Helping patients develop a deeper understanding of their symptoms can normalize the symptoms, which leads to relief for some individuals (59). Increasing knowledge about mental illness can be seen as an intervention in itself, which is consistent with the transtheoretical model of behavior change (i.e., being aware of the current stage of change) (60, 61).

*Session 2* focuses on behavior activation, which can help the patient identify triggers and subsequent avoidance behaviors. The therapist assists the patient in overcoming avoidance of certain situations and together with the patient identifies pleasurable activities based on their values, and encourages the patient to schedule and engage in these activities. Additionally, the patient may also identify specific thoughts and feelings that are related to the traumatic events that are facilitating either cognitive or behavioral avoidance. Participants are encouraged to set goals that are consistent with their values and work toward accomplishing these goals (i.e., taking their children to the zoo; driving alone at night, etc.). Behavior activation is an important step in this treatment because it acts as a proxy for exposure in helping a patient to overcome avoidance behaviors. It has also been shown to be an effective treatment for depression, which is often comorbid with PTSD symptoms (62). Avoidance of pleasurable activities may also maintain symptoms by preventing the reinforcement of the patient's natural environment.

*Session 3* introduces the idea and practice of mindfulness, which is targeted to address the arousal symptoms that are the hallmark of trauma-related mental health symptoms. Awareness and acceptance of trauma-related thoughts and feelings may serve as an indirect mechanism of cognitive-affective exposure. This may be especially useful for individuals with trauma-related symptoms as it may help reduce arousal, and foster emotion regulation. Regular mindfulness practice has been shown to decrease physiological arousal (63). Finally, *Session 4* reviews mindfulness concepts and practice as well as focuses on future goals that the patient would like to accomplish. Relapse prevention is also discussed in this session as a way to help the patient identify symptoms that would necessitate a return to treatment in the future.

### Statistical Analysis Plan

Demographic data was summarized for *N* = 32 women who had screening and/or baseline PCL data with means and standard deviations for continuous variables and frequencies and percentages for categorical variables. Demographic variables were compared (i) between sites, (ii) between those who completed at least one intervention session compared to none, (iii) between those missing one or more intervention session with those who completed all intervention sessions, and (iv) between those missing their 10–14 week follow-up and those who completed their 10–14 week follow-up; differences were tested with two-sample *t*-tests or Fisher's exact tests, as appropriate. There were no significant differences in demographics for any of these comparisons, so models did not account for site and missing longitudinal data was assumed to be missing completely at random.

Continuous longitudinal assessment variables (i.e., EPDS, PSS, and PCL) were modeled as repeated measures outcomes using generalized estimating equations models (GEEs) with exchangeable correlation structures; binary outcomes (e.g., EPDS >10) were modeled in this framework with a logit link. Adjusted models included number of completed treatment visits. Gestational age at baseline was considered as a covariate but as it did not have any significant effect on the significance or direction of any of the adjusted results it was not ultimately included in the models.

When arousal symptoms were analyzed, a PCL item score of ≥3 was used to determine whether an item was endorsed. DSM-IV-TR criteria for meeting the arousal cluster require endorsement of 2 out of 5 items. Severity was calculated by summing the scores of the five arousal items. Total number of ACEs experienced and total TLEQ score were considered as important predictors of these longitudinal psychological outcomes, and it was hypothesized that they would predict engagement in the study treatment (44, 45). First, each of these were tested with one-way ANOVAs or two-sample *t*-tests, as appropriate, to determine if they were significant predictors of number of visits completed or completing the intervention. Then, each of these continuous variables was added as a predictor in the GEE models to determine if they had significant effects on mean levels of outcomes over time. To determine if total ACE or total TLEQ had an effect on outcomes over time, an interaction was tested in each model between each of these predictors with time.

Data were analyzed with an intent-to-treat analysis; participant data was included even if all study visits or measures were not completed. Adjusted models accounted for number of completed visits. A significance level of alpha = 0.05 was used for all tests, and effect sizes are reported with Wald statistics (W) from robust standard errors in GEE models, *F* statistics for ANOVAs, and *t* statistics for two-sample *t*-tests. Statistical analyses were conducted in R version 4.0.5 (64).

### Participants

Demographic information is presented in Table 1. The sample was comprised of 32 pregnant women with an average gestational age of 18.5 weeks at the time of enrollment. The sample was overwhelmingly non-Caucasian, single, and reported very low income (under $25,000).

**Table 1:** Baseline demographic characteristics for all participants and by attendance.

**Variable**	**Overall (*N* = 32)**	**Missing 1+ intervention session (*N* = 19)**	**Completed all 4 sessions (*N* = 13)**	***P*-value**
Age m (SD)	26.0 (5.2)	26.5 (5.4)	25.2 (5.1)	0.524
**Race**				
Black/African American	25 (78.1%)	16 (84.2%)	9 (69.2%)	0.564
Caucasian	4 (12.5%)	2 (10.5%)	2 (15.4%)	
Other	3 (9.4%)	1 (5.3%)	2 (15.4%)	
Number of lifetime pregnancies	2.9 (1.5)	3.2 (1.8)	2.6 (1.0)	0.274
Number of children	1.1 (1.3)	1.3 (1.6)	0.8 (0.7)	0.195
**Marital status**				
Single/separated	27 (84.4%)	16 (84.2%)	11 (84.6%)	1
Married/domestic partner	5 (15.6%)	3 (15.8%)	2 (15.4%)	
**Income**				
$25 + K	4 (12.5%)	1 (5.3%)	3 (23.1%)	0.279
$25K or less	28 (87.5%)	18 (94.7%)	10 (76.9%)	
**Education**				
High school graduate or less	17 (53.1%)	12 (63.2%)	5 (38.5%)	0.280
Associate/some college or more	15 (46.9%)	7 (36.8%)	8 (61.5%)	
**Baseline PCL score**	56.4 (8.8)	56.0 (7.9)	57.0 (10.3)	0.770
**Baseline EPDS score**	13.3 (5.2)	13.3 (5.2)	13.3 (5.2)	0.225

**Figure 1:**
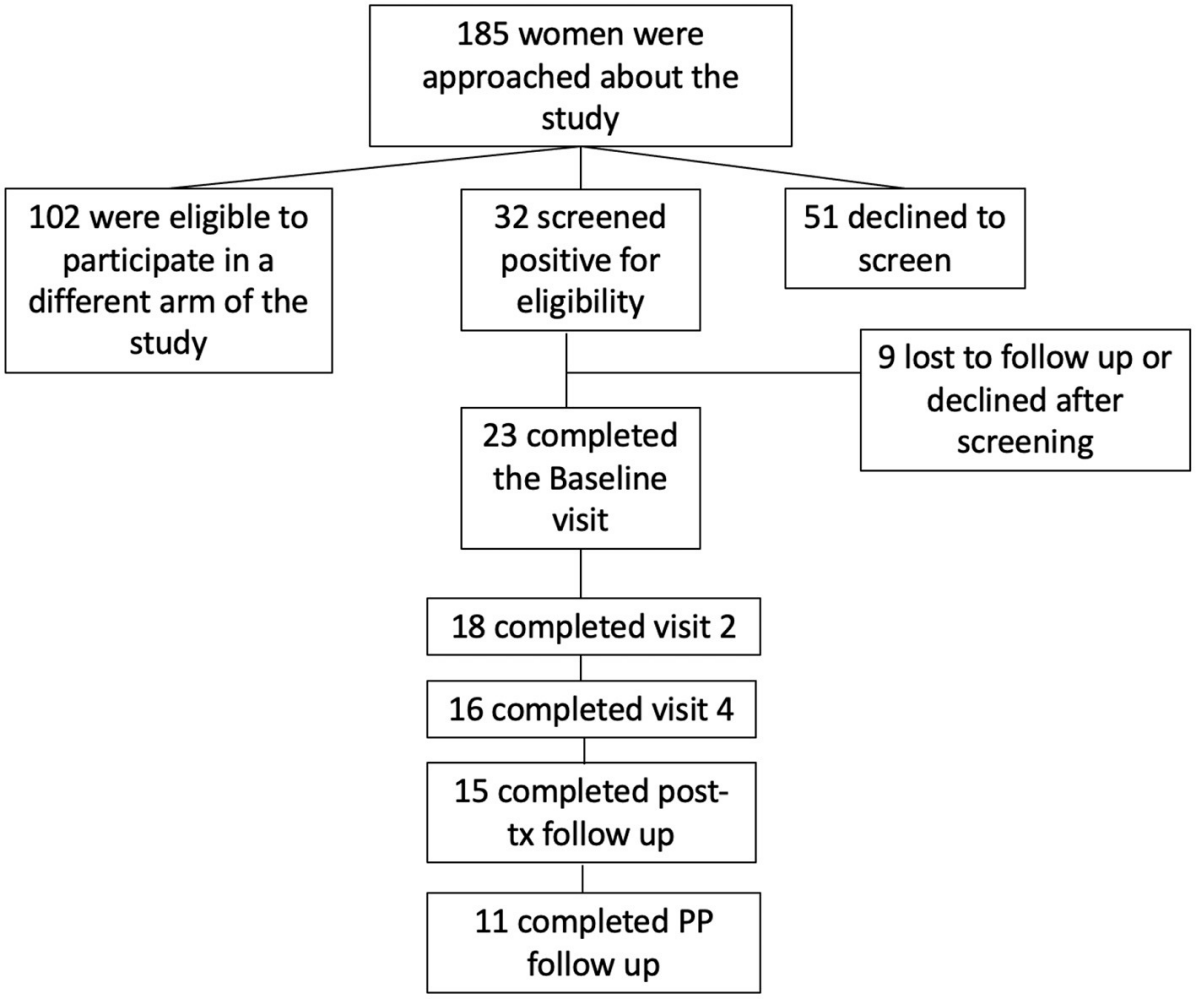
Participant enrollment flowchart.

One hundred eighty five (185) women were approached about the study, 32 screened positive for eligibility and consented to participate, and 23 completed the baseline visit. Of those 23, 18 completed measures at visit 2; 16 at visit 4, 15 at post-treatment follow-up, and 11 at the final follow-up, 10–14 weeks post-partum. See Figure 1. We collected delivery data on 25 participants. Adjusted models described below account for the number of completed treatment visits. There were no significant differences in demographics between women who enrolled and then failed to follow-up or dropped out after the Baseline visit (data not shown).

## Results

### PTSD

**Figure 2:**
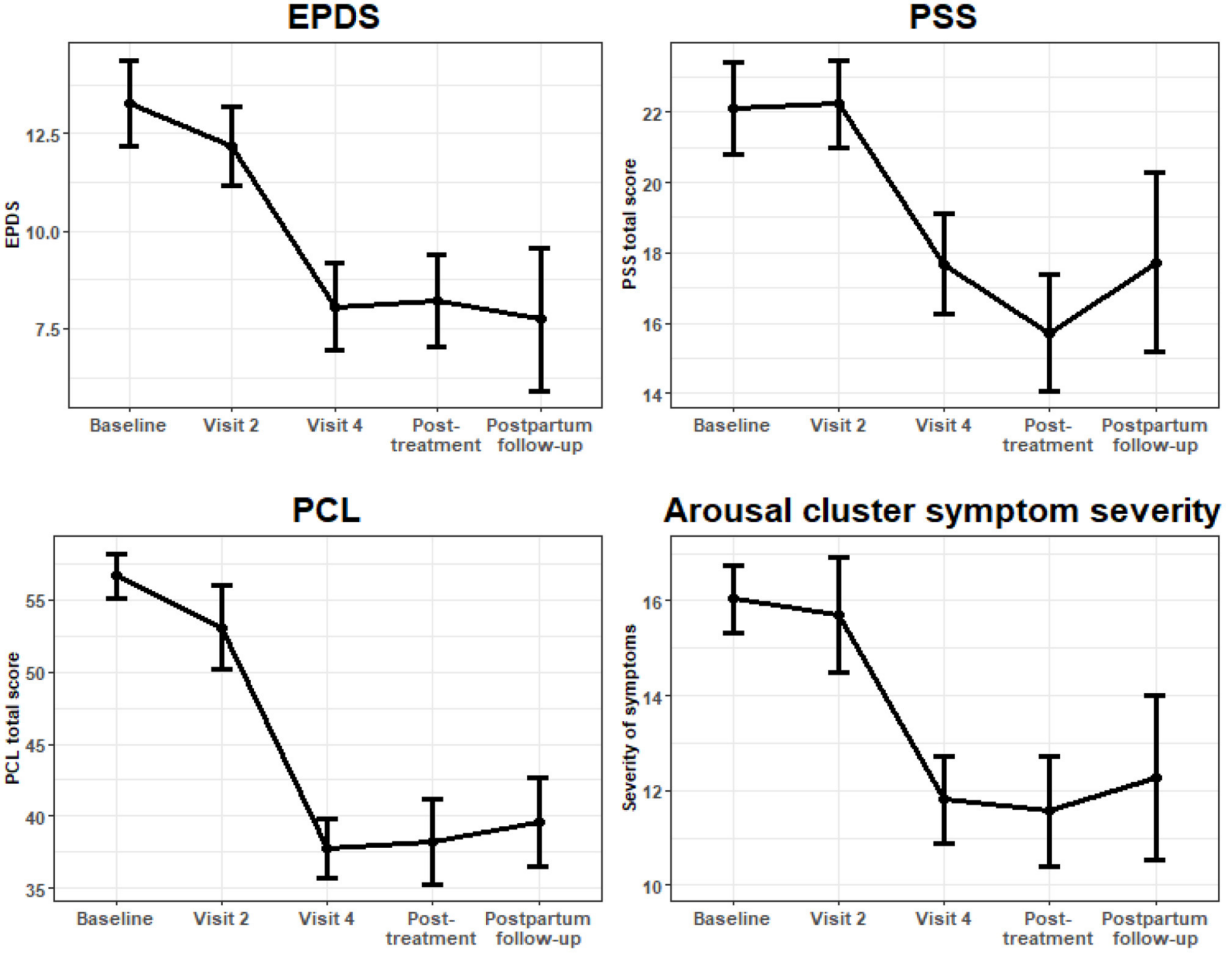
Longitudinal outcomes of symptom severity scales plotted with mean standard errors at each visit.

At baseline, mean PCL-C score was 56.4 (SD 8.9). At the final session of the intervention (visit 4), PCL had decreased an average of 20.27 points (95% CI: −25.62, −14.92, *P* < 0.001, W = −7.43) after adjusting for number of completed treatment visits. This effect remained at both follow-up time points; with PCL-C score remaining significantly lower than at baseline at the post-treatment follow-up (−19.81, 95% CI: −25.30, 14.32, *P* < 0.001, W = −7.08) and at post-partum follow-up at 10–14 weeks post-partum (−19.56, 95% CI: −25.30, −14.32, *P* < 0.001, W = −6.85). See Figure 2.

Given our specific interest in the relationship of arousal in PTSD to adverse pregnancy outcomes, (22) we investigated change in the number of arousal symptoms reported. Using number of arousal symptoms endorsed as a continuous variable, at the baseline visit, the mean (SD) number of arousal symptoms endorsed was 3.3 (1.4). By the final intervention session (visit 4), the number of arousal symptoms was on average 1.55 points lower (95% CI: −2.35, −0.75, *P* < 0.001, W = −3.80), and this effect remained significant at the post-treatment follow-up visit (−1.83; 95% CI: −2.72, −0.94, *P* < 0.001, W = −4.03), as well as at the post-partum follow-up visit (−1.90, 95% CI: −2.82, −0.98, *P* < 0.001, W = −4.05).

Similarly, when we investigated the change in severity of the arousal symptoms, we found significant and sustained decreases in arousal symptom severity at visit 4 (−5.04; 95% CI: −7.06, −3.02, *P* < 0.001, W = 4.90), post-treatment (−5.45; 95% CI: −7.75, −3.14, *P* < 0.001, W = −4.63), and post-partum follow-ups (−5.05, 95% CI: −8.32, −1.77, W = 3.02). For these analyses, severity of arousal symptoms was determined by the sum of scores on PCL-IV items corresponding to cluster D (items 13–17).

Lastly, we investigated changes in whether criteria were met for the arousal symptoms cluster of the PTSD diagnosis; 2 out of 5 arousal cluster symptoms. Compared to baseline, at visit 4 there was a significant decrease in the likelihood of meeting criteria for the arousal symptom cluster (D) (aOR = 0.16, 95% CI: 0.04, 0.69, *P* = 0.014, W = −2.32), and this change was sustained at both follow-up time points (post-treatment follow-up: aOR = 0.12, 95% CI: 0.02, 0.59, *P* = 0.009, W = −2.57; post-partum follow-up: aOR = 0.05, 95% CI: 0.01, 0.37, *P* = 0.003, W = −2.95).

### Depression

At baseline, mean EPDS score was 13.3 (*SD* = 5.2). Over the course of the intervention and follow-up period, there were significant sustained decreases in depression score, as measured by the EPDS. By visit 4, the adjusted mean difference in EPDS score compared to baseline was −4.81 (95% CI: −7.55, −2.06, *P* = 0.001, W = −3.23), and results remained consistent at the post-treatment follow-up (−4.54, 95% CI: −7.29, −1.79, *P* = 0.001, W = −3.10) and post-partum follow-up (−5.46, 95% CI: −9.15, −1.77, *P* = 0.004, W = −2.81). See Figure 1. Similar results were found when modeling EPDS as a binary outcome (EPDS >10; EPDS >13). After adjustment, there were significantly lower odds of having an EPDS >10 at visit 4 (*P* = 0.003, W = −2.77), post-treatment (*P* = 0.040, W = −2.07) and post-partum follow-up (*P* = 0.013, W = −2.47), as well as significantly lower odds of having an EPDS score >13 at these visits (*P* = 0.017, W = −2.29; *P* = 0.006 W = −2.80; *P* = 0.033, W = −2.17, respectively).

### Number of Sessions Required for Improvement

We examined change in score for the PCL, EPDS, PSS, and number of arousal symptoms at each treatment session to determine whether there was a treatment effect by session. For all measures except the PCL, treatment effects did not become evident until session 4. For PCL scores, improvement was seen beginning at session 2 in both unadjusted models and those models that adjusted for the number of completed treatment visits (PCL Visit 2, adjusted estimate = −5.18 95% CI: −9.85, −0.52, *P* = 0.029; W = −2.34).

### Lifetime Stress Effects

Neither ACE nor TLEQ scores had a significant impact on treatment engagement, as measured by number of completed visits (ACE, *P* = 0.446, *F* = 0.61; TLEQ, *P* = 0.094, *F* = 3.00; See Tables 2, 3). Similarly, neither ACE nor TLEQ scores were related to treatment dropout after session 1, comparing those completing 0–3 sessions to those completing all four sessions (ACE, *P* = 0.883, *t* = 0.15, df = 14.48; TLEQ, *P* = 0.059, *t* = −1.98, df = 24.23).

**Table 2:** Total ACE and number of treatment sessions attended.

**Number follow-up visits**	***N***	**Mean (SD) total ACE**	***P* value**
0	2	6.50 (3.54)	0.446
1	2	3.50 (2.12)	
2	3	4.33 (2.08)	
3	2	3.50 (2.12)	
4	13	4.31 (1.80)	

*Differences tested with one-way ANOVAs or two-sample t-tests, as appropriate*.

**Table 3:** Total TLEQ and number of treatment sessions attended.

**Number follow-up visits**	***N***	**Mean (SD) total TLEQ**	***P* value**
0	11	6.91 (3.30)	0.094
1	2	7.50 (0.71)	
2	2	7.00 (1.41)	
3	2	6.50 (4.95)	
4	13	9.23 (3.30)	

*Differences tested with one-way ANOVAs or two-sample t-tests, as appropriate*.

Finally, we tested separate interactions between both ACE and TLEQ and time to determine whether an individual's ACE or TLEQ score affected the trajectory of improvement longitudinally over the four follow-up periods for depression, perceived stress, and PTSD symptoms. Our findings indicate a relationship between TLEQ and EPDS, analyzed continuously, (unadjusted and adjusted models, *P* < 0.001; global *Z*-test for interaction 4.35, 4.44) such that higher TLEQ scores were associated with increases in depression symptoms from pregnancy to post-partum.

Similarly, ACE scores significantly affected EPDS trajectory over time in both unadjusted (*P* = 0.002) and adjusted (*P* = 0.003) models; with an association showing higher ACE scores were associated with more depressive symptoms in both the unadjusted (*P* = 0.002, Z = 3.44) and adjusted (*P* = 0.003, Z = 3.40) models. However, this latter result should be interpreted with caution since the range of ACE scores was truncated at the post-partum follow-up timepoint (range, 2–6), post-partum *N* = 11, limiting the generalizability of this result.

## Discussion

In a novel study testing the efficacy of a brief treatment for clinically meaningful PTSD in a sample of non-treatment seeking, majority Black pregnant women we found that PTSD symptoms, depression, and stress all significantly decreased in response to the intervention. Strikingly, these benefits persisted into the post-partum period and were sustained at 10–14 weeks after birth. The intervention was designed to address the specific detrimental effects of psychophysiological arousal that is characteristic of full PTSD or pPTSD, and our findings suggest that this was successful. The total number and severity of arousal symptoms, and the proportion of participants meeting criteria for the arousal cluster diagnosis in DSM-IV-TR were all significantly reduced.

Psychotherapeutic treatment for PTSD has been established as a safe option for pregnant individuals, (65, 66) despite commonly held beliefs that discussing trauma may be stressful and thus detrimental to an expectant mother or her child (67). However, few studies include pregnant women in research investigating psychotherapeutic treatments for PTSD, (68, 69) and even fewer specifically target perinatal PTSD or pPTSD (42, 66).

This gap in the literature represents a major oversight given the risks of untreated PTSD in pregnancy, the potential risk that it poses for offspring, and given that the prevalence of PTSD in pregnancy approaches or surpasses the prevalence rates of other major pregnancy complications that are associated with significant morbidity and mortality (70). The findings of this treatment study indicate that even a brief and short-term psychotherapeutic intervention can have clinically meaningful benefits for pregnant patients. That these effects persist into the post-partum period has implications for the benefits of trauma-focused therapy on post-partum maternal and family functioning and the potential for improved longitudinal child outcomes (71–74).

Prenatal interventions for PTSD have largely been neglected in favor of screening and treatment efforts for post-partum depression (42, 66). Perinatal PTSD is undetected and untreated in the vast majority of women, (10, 75) and pPTSD may be even more overlooked. However, the neglect of PTSD during pregnancy may be short sighted given the frequency of comorbid diagnoses of PTSD and depression. For example, based on recent studies among trauma-exposed perinatal women, as many as 65–85% of those with depression also have PTSD (76) or pPTSD (77). This comorbidity suggests that a focus on depression risk alone is too narrow. The treatment tested in this study indicated benefit for both symptoms of PTSD as well as depression, suggesting that it may be possible to combine treatment efforts in short-term psychotherapies and generate lasting benefit for perinatal women.

Our investigation to determine the minimum number of treatment sessions necessary to see symptom improvement suggested that depression and perceived stress required a complete course of treatment, while for PTSD symptoms, benefits were realized by session 2. These findings are perhaps unsurprising since the treatment was designed to address PTSD symptoms first and psychoeducation about trauma, maintaining features, and treatment planning are addressed in session 1. Session 2 focused on behavior activation and set a plan for participants to enact their behavior activation goals prior to the next session, and session 3 focused on an introduction to mindfulness. As it is well-established that behavior activation (78) and mindfulness-based interventions (79) are effective treatments for depression (80), it is not surprising to see improvement in depression symptoms after these topics are addressed in the study treatment.

Psychiatric treatment initiation and follow-up during pregnancy is known to be challenging, with high rates of drop-out (45). This effect may be even more heightened in treatment for PTSD as concerns have been noted about the tolerability of exposure therapies, (81, 82) despite empirical evidence suggesting that exposure therapy for PTSD is not associated with greater risk for treatment drop-out (83). We nonetheless explored attrition based on prior experience of trauma. Consistent with prior literature, we found that neither ACE nor TLEQ scores conferred additional risk of failure to initiate treatment, and neither were associated with failure to complete the four-session course of psychotherapy. This finding is especially noteworthy since our sample was non-treatment seeking women, who were recruited, screened and offered psychotherapy in the course of their standard prenatal care. Our results highlight the urgent need for effective psychotherapies to address prenatal PTSD or pPTSD.

Our study included majority low-income, low-resourced patients with mental health concerns, who have been shown to be less likely to obtain mental health care in the traditional setting (84). Despite the significant impairment (3) and chronicity (85) associated with both PTSD and pPTSD, a significant number of individuals and especially minoritized individuals with these disorders never seek or receive any treatment for their symptoms (86). Prior literature suggests that the major barrier to seeking treatment is access and knowledge about where to seek treatment, (86) which may have also been the case amongst the women enrolled in this study. Given that the majority who initiated treatment completed all four sessions, our results suggest four sessions of psychotherapy is acceptable and feasible for non-treatment seeking pregnant women. As we did not collect data specifically on acceptability and feasibility, this inference should be interpreted with caution. Our study offered the intervention either in the prenatal care clinic or in outpatient offices of psychologists located several blocks away; therefore future research should explore acceptability of treatment offered in different settings that are convenient for the patient, including home-based treatments offered online (87–89).

This study is not without limitations. Our sample size is small, without a control group, and limited to a cohort of women who are demographically homogenous. However, given high levels of trauma and poor access to mental health services in low-income, majority Black communities (8, 9, 90) the findings may be generalizable to pregnant women in other similar communities outside of Philadelphia and St. Louis (9). Given the small sample size, we were unable to investigate the relationship between improvement in PTSD and pPTSD symptoms and preterm birth or other pregnancy or birth outcomes. We are also limited by the reliance on a self-report measure based on prior diagnostic criteria for PTSD (91). However, a recent study (92) suggests substantial agreement between the PCL-C used in this study and newer version of the PCL developed for use with DSM-5 (93). With pilot data that supports our hypotheses that our treatment can improve symptoms in a lasting way, future studies should aim to offer these clinical services to more women to reach stronger conclusions about effects of mental health services offered during pregnancy to positively impact pregnancy and birth outcomes.

## Conclusions

Our findings highlight the benefit of a course of brief, short-term psychotherapy designed to alleviate symptoms of PTSD or pPTSD in a cohort of non-treatment-seeking, mostly Black low-income pregnant women. Given the high prevalence of PTSD or pPTSD among trauma-exposed pregnant women, the frequency of comorbid depression, and the lack of existing acceptable treatment options for this population, our study suggests that brief treatments may be sufficient and acceptable and result in lasting change with implications for improved functioning in the post-partum period. Given the promising outcome of this brief perinatal intervention for non-treatment seeking women, future research should expand on and replicate the work in a randomized controlled trial to establish the effectiveness of the intervention and improve the generalizability of our findings. That a brief trauma-focused intervention can have such lasting benefit for women, and by extension their families, children, and communities warrants further exploration to make this approach a mainstay of perinatal care.

## Data Availability Statement

The raw data supporting the conclusions of this article will be made available by the authors, without undue reservation.

## Ethics Statement

This study was conducted following ethical guidelines and as such was approved by the IRBs of both the University of Pennsylvania and the Washington University in St. Louis. The patients/participants provided their written informed consent to participate in this study.

## Author Contributions

SK and NE designed the study. RK organized the database. RJ and MS performed and supervised the statistical analyses. SK wrote the first draft of the manuscript. RJ, LH, NE, RW, and SK contributed to writing and editing sections of the manuscript. All authors contributed to manuscript revisions, and read and approved the submitted version.

## Funding

This study was funded by NIMH K23MH102360 (SK) and NIMH K23MH107831 (LH).

## Conflict of Interest

The authors declare that the research was conducted in the absence of any commercial or financial relationships that could be construed as a potential conflict of interest.

## Publisher's Note

All claims expressed in this article are solely those of the authors and do not necessarily represent those of their affiliated organizations, or those of the publisher, the editors and the reviewers. Any product that may be evaluated in this article, or claim that may be made by its manufacturer, is not guaranteed or endorsed by the publisher.

## References

[1] Bothe T Jacob J Kröger C. How expensive are post-traumatic stress disorders? Estimating incremental health care and economic costs on anonymised claims data. Eur J Health Econ. (2020) 21:917–30. 10.1007/s10198-020-01184-x 32458163 PMC7366572

[2] Pietrzak RH Goldstein RB Southwick SM. Medical comorbidity of full and partial post-traumatic stress disorder in US adults: results from wave 2 of the national epidemiologic survey on alcohol and related conditions. Psychosom Med. (2011) 73:697–707. 10.1097/PSY.0b013e3182303775 21949429 PMC3188699

[3] Breslau N Lucia VC Davis GC. Partial PTSD vs. full PTSD: an empirical examination of associated impairment. Psychol Med. (2004) 34:1205–14. 10.1017/S0033291704002594 15697047

[4] Mylle J Maes M. Partial post-traumatic stress disorder revisited. J Affect Disord. (2004) 78:37–48. 10.1016/S0165-0327(02)00218-5 14672795

[5] Hepp U Gamma A Milos G Eich D Ajdacic-Gross V Rössler W. Prevalence of exposure to potentially traumatic events and PTSD. The Zurich cohort study. Eur Arch Psychiatry Clin Neurosci. (2006) 256:151–8. 10.1007/s00406-005-0621-7 16267635

[6] Stein MB Walker JR Hazen AL Forde DR. Full and partial post-traumatic stress disorder: findings from a community survey. Am J Psychiatry. (1997) 154:1114–9. 10.1176/ajp.154.8.1114 9247398

[7] Zhang W Ross J. Post-traumatic stress disorder in callers to the Anxiety Disorders Association of America. Depress Anxiety. (2004) 19:96–104. 10.1002/da.10138 15022144

[8] Gillespie CF Bradley B Mercer K Smith AK Conneely K Gapen M. Trauma exposure and stress-related disorders in inner city primary care patients. Gen Hosp Psychiatry. (2009) 31:505–14. 10.1016/j.genhosppsych.2009.05.003 19892208 PMC2785858

[9] Powers A Woods-Jaeger B Stevens JS Bradley B Patel MB Joyner A. Trauma, psychiatric disorders, and treatment history among pregnant African American women. Psychol Trauma. (2020) 12:138–46. 10.1037/tra0000507 31464464 PMC6986992

[10] Yildiz PD Ayers S. The prevalence of post-traumatic stress disorder in pregnancy and after birth: a systematic review and meta-analysis. J Affect Disord. (2017) 208:634–45. 10.1016/j.jad.2016.10.009 27865585

[11] Rowe H Sperlich M Cameron H. A quasi-experimental outcomes analysis of a psychoeducation intervention for pregnant women with abuse-related post-traumatic stress. J Obstet Gynecol Neonatal Nurs. (2014) 43:282–93. 10.1111/1552-6909.12312 24754455 PMC4318682

[12] Seng JS Sperlich M Low LK Ronis DL Muzik M. Childhood abuse history, post-traumatic stress disorder, post-partum mental health, and bonding: a prospective cohort study. J Midwifery Womens Health. (2013) 58:57–68. 10.1111/j.1542-2011.2012.00237.x 23374491 PMC3564506

[13] Forray A Mayes LC Magriples U. Prevalence of post-traumatic stress disorder in pregnant women with prior pregnancy complications. J Matern-Fetal Neonatal Med. (2009) 22:522–7. 10.1080/14767050902801686 19488936 PMC4109276

[14] Alim TN Charney DS. An overview of post-traumatic stress disorder in African Americans. J Clin Psychol. (2006) 62:801–13. 10.1002/jclp.20280 16703601

[15] Loveland Cook CA Flick LH Homan SM Campbell C McSweeney M Gallagher ME. Post-traumatic stress disorder in pregnancy: prevalence, risk factors, and treatment. Obstet Gynecol. (2004) 103:710–7. 10.1097/01.AOG.0000119222.40241.fb 15051563

[16] Seng JS Oakley DJ Sampselle CM Killion C Graham-Bermann S. Post-traumatic stress disorder and pregnancy complications. Obstet Gynecol. (2001) 97:17–22. 10.1097/00006250-200101000-00004 11152900

[17] Shaw Jonathan G Asch Steven M Kimerling R Frayne SM Shaw KA Phibbs CS. Post-traumatic stress disorder and risk of spontaneous preterm birth. Obstet Gynecol. (2014) 124:1111–9 10.1097/AOG.0000000000000542 25415162

[18] Rondo PHC Ferreira RF Nogueira F Ribeiro MCN Lobert H Artes R. Maternal psychological stress and distress as predictors of low birth weight, prematurity and intrauterine growth retardation. Eur J Clin Nutr. (2003) 57:266–72. 10.1038/sj.ejcn.1601526 12571658

[19] Dole N Savitz DA Hertz-Picciotto I Siega-Riz AM McMahon MJ Buekens P. Maternal stress and preterm birth. Am J Epidemiol. (2003) 157:14–24. 10.1093/aje/kwf176 12505886

[20] Wadhwa P. Psychoneuroendocrine processes in human pregnancy influence fetal development and health. Psychoneuroendocrinology. (2005) 30:724–43. 10.1016/j.psyneuen.2005.02.004 15919579

[21] Kurki T Hiilesmaa V Raitasalo R Mattila H Ylikorkala O. Depression and anxiety in early pregnancy and risk for preeclampsia. Obstet Gynecol. (2000) 95:487–90. 10.1097/00006250-200004000-00003 10725477

[22] Yonkers KA Smith MV Forray A Epperson CN Costello D Lin H. Pregnant women with post-traumatic stress disorder and risk of preterm birth. JAMA Psychiatry. (2014) 71:897–904. 10.1001/jamapsychiatry.2014.558 24920287 PMC4134929

[23] Zuckerman B Bauchner H Parker S Cabral H. Maternal depressive symptoms during pregnancy, and newborn irritability. J Dev Behav Pediatr. (1990) 11:190–4. 10.1097/00004703-199008000-00006 2212032

[24] Huot RL Brennan PA Stowe ZN Plotsky PM Walker EF. Negative affect in offspring of depressed mothers is predicted by infant cortisol levels at 6 months and maternal depression during pregnancy, but not post-partum. Ann N Y Acad Sci. (2004) 1032:234–6. 10.1196/annals.1314.028 15677418

[25] O'Connor TG Heron J Golding J Beveridge M Glover V. Maternal antenatal anxiety and children's behavioural/emotional problems at 4 years. Br J Psychiatry. (2002) 180:502–8. 10.1192/bjp.180.6.502 12042228

[26] O'Donnell KJ Glover V Jenkins J Browne D Ben-Shlomo Y Golding J. Prenatal maternal mood is associated with altered diurnal cortisol in adolescence. Psychoneuroendocrinology. (2013) 38:1630–8. 10.1016/j.psyneuen.2013.01.008 23433748 PMC3695029

[27] Yehuda R. Intergenerational transmission of trauma effects: putative role of epigenetic mechanisms. World Psychiatry. (2018) 17:243–57. 10.1002/wps.20568 30192087 PMC6127768

[28] Smith MV Poschman K Cavaleri MA Howell HB Yonkers K. Symptoms of post-traumatic stress disorder in a community sample of low-income pregnant women. Am J Psychiatry. (2006) 163:881–4. 10.1176/ajp.2006.163.5.881 16648330

[29] Kornfield SL Moseley M Appleby D McMickens CL Sammel MD Epperson CN. Post-traumatic symptom reporting and reported cigarette smoking during pregnancy. J Womens Health. (2017) 26:662–9. 10.1089/jwh.2016.5928 28437216 PMC5512338

[30] Dailey DE Humphreys JC Rankin SH Lee K. An exploration of lifetime trauma exposure in pregnant low-income African-American women. Matern Child Health J. (2011) 15:410–8. 10.1007/s10995-008-0315-7 18253820 PMC3150846

[31] Morgan CA Grillon C Southwick SM Davis M Charney DS. Fear-potentiated startle in post-traumatic stress disorder. Biol Psychiatry. (1995) 38:378–85. 10.1016/0006-3223(94)00321-S 8547457

[32] Selye H. The Stress of Life. New York, NY: McGraw-Hill (1956).

[33] Challis JRG Sloboda D Matthews SG Holloway A Alfaidy N Patel FA. The fetal placental hypothalamic–pituitary–adrenal (HPA) axis, parturition and post-natal health. Mol Cell Endocrinol. (2001) 185:135–44. 10.1016/S0303-7207(01)00624-4 11738803

[34] Schrader C. A review of PTSD and current treatment strategies. Mo Med. (2021) 118:546–51. 34924624 PMC8672952

[35] Tew J Klaus J. The behavioral health laboratory: building a stronger foundation for the patient-centered medical home. Fam Syst Health. (2010) 28:130–45. 10.1037/a0020249 20695671

[36] Wolk CB Last BS Livesey C Oquendo MA Press MJ Mandell DS. Addressing common challenges in the implementation of collaborative care for mental health: the penn integrated care program. Ann Fam Med. (2021) 19:148–56. 10.1370/afm.2651 33685876 PMC7939709

[37] Hoeft TJ Stephens KA Vannoy SD Unützer J. Interventions to treat post-traumatic stress disorder in partnership with primary care: a review of feasibility and large randomized controlled studies. Gen Hosp Psychiatry. (2019) 60:65–75. 10.1016/j.genhosppsych.2019.05.008 31349204 PMC7592634

[38] Rauch S Cigrang J Austern D Evans A. Expanding the reach of effective PTSD treatment into primary care: prolonged exposure for primary care. Focus. (2017) 15:406–10. 10.1176/appi.focus.20170021 31975871 PMC6519517

[39] Cigrang JA Rauch SA Mintz J Brundige AR Mitchell JA Najera E. Moving effective treatment for post-traumatic stress disorder to primary care: a randomized controlled trial with active duty military. Fam Syst Health. (2017) 35:450–62. 10.1037/fsh0000315 29283612

[40] Stevens NR Miller ML Soibatian C Otwell C Rufa AK Meyer DJ. Exposure therapy for PTSD during pregnancy: a feasibility, acceptability, and case series study of narrative exposure therapy (NET). BMC Psychol. (2020) 8:130. 10.1186/s40359-020-00503-4 33298159 PMC7727253

[41] Hantsoo L Kornfield S Anguera MC. Inflammation: a proposed intermediary between maternal stress and offspring neuropsychiatric risk. Biol Psychiatry. (2019) 85:97–106. 10.1016/j.biopsych.2018.08.018 30314641 PMC6309506

[42] Nillni YI Mehralizade A Mayer L. Treatment of depression, anxiety, and trauma-related disorders during the perinatal period: a systematic review. Clin Psychol Rev. (2018) 66:136–48. 10.1016/j.cpr.2018.06.004 29935979 PMC6637409

[43] Hantsoo L Podcasy J Sammel M Epperson CN Kim DR. Pregnancy and the acceptability of computer-based vs. traditional mental health treatments. J Womens Health. (2017) 26:1106–13. 10.1089/jwh.2016.6255 28426287 PMC5651968

[44] Kramer MS Seguin L Lydon J Goulet L. Socio-economic disparities in pregnancy outcome: why do the poor fare so poorly? Paediatr Perinat Epidemiol. (2000) 14:194–210. 10.1046/j.1365-3016.2000.00266.x 10949211

[45] Kornfield SL Kang-Yi CD Mandell DS. Predictors and patterns of psychiatric treatment dropout during pregnancy among low-income women. Matern Child Health J. (2018) 22:226–36. 10.1007/s10995-017-2394-9 29143169 PMC5821232

[46] Munro A George RB Mackinnon SP Rosen NO. The association between labour epidural analgesia and post-partum depressive symptoms: a longitudinal cohort study. Can J Anaesth. (2021) 68:485–95. 10.1007/s12630-020-01900-4 33403538

[47] Blanchard EB Jones-Alexander J Buckley TC. Psychometric properties of the PTSD checklist (PCL). Behav Res Ther. (1996) 34:669–73. 10.1016/0005-7967(96)00033-2 8870294

[48] Cohen S Kamarck T. A global measure of perceived stress. J Health Soc Behav. (1983) 24:385–96. 10.2307/2136404 6668417

[49] Cox JL Holden JM. Detection of post-natal depression. Development of the 10-item Edinburgh post-natal depression scale. Br J Psychiatry. (1987) 150:782–6. 10.1192/bjp.150.6.782 3651732

[50] Murray D Cox JL. Screening for depression during pregnancy with the edinburgh depression scale (EDDS). J Reprod Infant Psychol. (1990) 8:99–107. 10.1080/02646839008403615

[51] Murray L Carothers AD. The validation of the edinburgh post-natal depression scale on a community sample. Br J Psychiatry. (1990) 157:288–90. 10.1192/bjp.157.2.288 2224383

[52] Weathers FW Litz BT Herman DS Huska JA Keane TM. The PTSD checklist: reliability, validity, & diagnostic utility. In: Paper Presented at the Annual Meeting of the ISTSS. San Antonio, TX (1993).

[53] Felitti VJ. Origins of the ACE study. Am J Prev Med. (2019) 56:787–9. 10.1016/j.amepre.2019.02.011 31104723

[54] Kubany ES Haynes SN Leisen MB Owens JA Kaplan AS Watson SB. Development and preliminary validation of a brief broad-spectrum measure of trauma exposure: the traumatic life events questionnaire. Psychol Assess. (2000) 12:210–24. 10.1037/1040-3590.12.2.210 10887767

[55] Bronson D Franco K. Post-traumatic stress disorder in primary care patients. Compr Ther. (2007) 33:208–15. 10.1007/s12019-007-8018-3 18025612

[56] Ford JD Ruzek J Niles B. Identifying and treating VA medical care patients with undetected sequelae of psychological trauma and post-traumatic stress disorder. NCP Clin Q. (1996) 6:77–82.

[57] Beck JS. Cognitive Behavior Therapy: Basics and Beyond. 2nd ed. New York, NY: Guilford Press (2011).

[58] Kabat-Zinn J. Mindfulness-based stress reduction (MBSR). Constr Hum Sci. (2003) 8:73–107.

[59] Resnick H Aciemo R Kilpatrick DG. Description of an early intervention to prevent substance abuse and psychopathology in recent rape victims. Behav Modif . (2005) 29:156–88. 10.1177/0145445504270883 15557482

[60] Prochaska JO DiClemente CC. The Transtheoretical Approach: Crossing Traditional Boundaries of Therapy. Homewood, IL: Dow Jones-Irwin (1984).

[61] Prochaska JO. The transtheoretical model of health behavior change. Am J Health Promot. (1997) 12:38–48. 10.4278/0890-1171-12.1.38 10170434

[62] Jakupcak M Roberts LJ Martell CR Mulick PS Michael S Reed S. A pilot study of behavioral activation for veterans with PTSD. J Trauma Stress. (2006) 19:387–91. 10.1002/jts.20125 16789005

[63] Baer RA. Mindfulness training as a clinical intervention: a conceptual and empirical review. Clin Psychol: Sci Pract. (2003) 10:125–43. 10.1093/clipsy.bpg015

[64] R Core Team. R: A Language Environment for Statistical Computing. Vienna: R Foundation for Statistical Computing (2021). Available online at: https://www.R-project.org/

[65] Baas MAM van Pampus MG Braam L Stramrood CAI de Jongh A. The effects of PTSD treatment during pregnancy: systematic review and case study. Eur J Psychotraumatol. (2020) 11:1762310. 10.1080/20008198.2020.1762310 33029304 PMC7473051

[66] Stevens NR Miller ML Puetz AK Padin AC Adams N. Psychological intervention and treatment for post-traumatic stress disorder during pregnancy: a systematic review and call to action. J Trauma Stress. (2021) 34:575–85. 10.1002/jts.22641 33340151

[67] Forgash C Leeds A Stramrood CAI. Case consultation: traumatized pregnant woman. J EMDR Pract Res. (2013) 7:45–9. 10.1891/1933-3196.7.1.45

[68] Blehar MC Spong C Grady C Goldkind SF Sahin L. Enrolling pregnant women: issues in clinical research. Womens Health Issues. (2013) 23:e39–45. 10.1016/j.whi.2012.10.003 23312713 PMC3547525

[69] Pawluski J. Pregnancy: a final frontier in mental health research. Arch Womens Ment Health. (2019) 22:831–2. 10.1007/s00737-019-00988-y 31289939

[70] Umesawa M. Epidemiology of hypertensive disorders in pregnancy: prevalence, risk factors, predictors and prognosis. Hypertens Res. (2017) 40:213–20. 10.1038/hr.2016.126 27682655

[71] Slomian J Honvo G Emonts P Reginster JV. Consequences of maternal post-partum depression: a systematic review of maternal and infant outcomes. Women's Health. (2019) 15:1–55. 10.1177/1745506519844044 31035856 PMC6492376

[72] Cross D Vance LA Kim YJ Ruchard AL Fox N Jovanovic T. Trauma exposure, PTSD, and parenting in a community sample of low-income, predominantly African American mothers and children. Psychol Trauma. (2018) 10:327–35. 10.1037/tra0000264 28481561 PMC5677577

[73] Oh W Muzik M McGinnis EW Hamilton L Menke RA Rosenblum KL. Comorbid trajectories of post-partum depression and PTSD among mothers with childhood trauma history: course, predictors, processes and child adjustment. J Affect Disord. (2016) 200:133–41. 10.1016/j.jad.2016.04.037 27131504 PMC4887316

[74] Posmontier B. Functional status outcomes in mothers with and without post-partum depression. J Midwifery Womens Health. (2008) 53:310–8. 10.1016/j.jmwh.2008.02.016 18586183 PMC2535808

[75] Dindo L Elmore A O'Hara M. The comorbidity of axis I disorders in depressed pregnant women. Arch Womens Ment Health. (2017) 20:757–64. 10.1007/s00737-017-0769-y 28842756 PMC5759963

[76] Grote NK Katon WJ Russo JE Lohr MJ Curran M Galvin E. A randomized trial of collaborative care for perinatal depression in socioeconomically disadvantaged women: the impact of comorbid post-traumatic stress disorder. J Clin Psychiatry. (2016) 77:1527–37. 10.4088/JCP.15m10477 28076671

[77] Seng JS Low LK Sperlich M Ronis DL. Prevalence, trauma history, and risk for post-traumatic stress disorder among nulliparous women in maternity care. Obstet Gynecol. (2009) 114:839–47. 10.1097/AOG.0b013e3181b8f8a2 19888043 PMC3124073

[78] Dimidjian S Martell CR Herman-Dunn R. Behavioral activation for depression. In: Barlow DH editor. Clinical Handbook of Psychological Disorders: A Step-by-Step Treatment Manual. New York, NY: The Guilford Press (2014). p. 353–93.

[79] Khoury B Lecomte T Fortin G Masse M Therien P Bouchard V. Mindfulness-based therapy: a comprehensive meta-analysis. Clin Psychol Rev. (2013) 33:763–71. 10.1016/j.cpr.2013.05.005 23796855

[80] Hofmann SG. Mindfulness-based interventions for anxiety and depression. Psychiatr Clin North Am. (2017) 40:739–49. 10.1016/j.psc.2017.08.008 29080597 PMC5679245

[81] Becker CB Zayfert C Anderson E. A survey of psychologists' attitudes towards and utilization of exposure therapy for PTSD. Behav Res Ther. (2004) 42:277–92. 10.1016/S0005-7967(03)00138-4 14975770

[82] Zayfert C Becker CB. Implementation of empirically supported treatments for PTSD: obstacles and innovations. Behav Ther. (2000) 23:161–8.

[83] Hembree EA Foa EB Dorfan NM Street GP Kowlaski J Tu X. Do patients drop out prematurely from exposure therapy for PTSD? J Trauma Stress. (2004) 16:555–62. 10.1023/B:JOTS.0000004078.93012.7d 14690352

[84] U.S. Department of Health and Human Services. Mental Health: Culture, Race, and Ethnicity: A Supplement to Mental Health: A Report of the Surgeon General. Rockville, MD: US Dept of Health and Human Services, Public Health Service, Office of the Surgeon General (2001). 20669516

[85] Cukor J Wyka K Jayasinghe N Difede J. The nature and course of subthreshold PTSD. J Anxiety Disord. (2010) 24:918–23. 10.1016/j.janxdis.2010.06.017 20655169

[86] Koenen KC Goodwin R Struening E Hellman F Guardino M. Post-traumatic stress disorder and treatment seeking in a national screening sample. J Trauma Stress. (2003) 16:5–16. 10.1023/A:1022051009330 12602647

[87] Strachan M Gros DF Yuen E Ruggiero KJ Foa EB. Home-based telehealth to deliver evidence-based psychotherapy in veterans with PTSD. Contemp Clin Trials. (2012) 33:402–9. 10.1016/j.cct.2011.11.007 22101225

[88] Langarizadeh M Tabatabaei MS Tavakol K Naghipour M Rostami A. Telemental health care, an effective alternative to conventional mental care: a systematic review. Acta Inform Med. (2017) 25:240–6. 10.5455/aim.2017.25.240-246 29284913 PMC5723163

[89] Fernandez E Woldgabreal Y Day A Pham T Gleich B. Live psychotherapy by video vs. in-person: a meta-analysis of efficacy and its relationship to types and targets of treatment. Clin Psychol Psychother. (2021) 10:1535–49. 10.1002/cpp.2594 33826190

[90] Brandow CL Swarbrick M. Improving black mental health: a collective call to action. Psychiatr Serv. (2021). 10.1176/appi.ps.202000894. [Epub ahead of print]. 34587786

[91] American Psychiatric Association. Diagnostic and Statistical Manual of Mental Disorders. 4th ed. Washington, DC: American Psychiatric Association (2000).

[92] LeardMann CA McMaster HS Warner S Esquivel AP Porter B Powell TM. Comparison of post-traumatic stress disorder checklist instruments from diagnostic and statistical manual of mental disorders, fourth edition vs. fifth edition in a large cohort of US military service members and veterans. JAMA Netw Open. (2021) 4:e218072. 10.1001/jamanetworkopen.2021.8072 33904913 PMC8080232

[93] American Psychiatric Association. Diagnostic and Statistical Manual of Mental Disorders. (5th ed). Washington, DC: American Psychiatric Association (2013). 10.1176/appi.books.9780890425596

